# Enhanced Photoluminescence of Gd_3_Al_4_GaO_12_: Cr^3+^ by Energy Transfers from Co-Doped Dy^3+^

**DOI:** 10.3390/nano12234183

**Published:** 2022-11-25

**Authors:** Yu Zhang, Xiang Li, Dahai Hu, Qier Sa, Xinran Wang, Fengxiang Wang, Kaixuan Wang, Xuelian Zhou, Zhiqiang Song, Yongfu Liu, Kefu Chao

**Affiliations:** 1 Inner Mongolia Key Laboratory of Physics and Chemistry of Functional Materials, College of Physics and Electronic Information, Inner Mongolia Normal University, Hohhot City 010022, China; 2 Inner Mongolia Engineering Research Center for Rare Earth Functional and New Energy Storage Materials, Hohhot City 010022, China; 3 Ningbo Institute of Materials Technology and Engineering, Chinese Academy of Sciences, Ningbo 315201, China

**Keywords:** Gd_3_Al_4_GaO_12_:Cr^3+^, Dy^3+^, phosphors, energy transfer, LEDs

## Abstract

LEDs for plant lighting have attracted wide attention and phosphors with good stability and deep-red emission are urgently needed. Novel Cr^3+^ and Dy^3+^ co-doped Gd_3_Al_4_GaO_12_ garnet (GAGG) phosphors were successfully prepared through a conventional solid-state reaction. Using blue LEDs, a broadband deep-red emission at 650–850 nm was obtained due to the Cr^3+ 4^T_2_ → ^4^A_2_ transition. When the Cr^3+^ concentration was fixed to 0.1 mol, the crystal structure did not change with an increase in the Dy^3+^ doping concentration. The luminous intensity of the optimized GAGG:0.1Cr^3+^, 0.01Dy^3+^ was 1.4 times that of the single-doped GAGG:0.1Cr^3+^. Due to the energy transfer from Dy^3+^ to Cr^3+^, the internal quantum efficiency reached 86.7%. The energy transfer from Dy^3+^ to Cr^3+^ can be demonstrated through luminescence spectra and fluorescence decay. The excellent properties of the synthesized phosphor indicate promising applications in the agricultural industry.

## 1. Introduction

Lighting is one important factors affecting plant growth. Photopigment P_R_ and P_FR_ mainly absorb deep-red light at 660–730 nm. P_R_ and P_FR_ play vital roles at all stages of plant growth and development, such as promoting seed germination, desiccating, stem growth, leaf expansion, shading and inducing effects, etc. [1,2,3,4]. However, traditional light sources, such as incandescent lamps, metal halide lamps, fluorescent lamps, and high-pressure sodium lamps, have the disadvantages of high costs and short lives. At present, the white LEDs existing in the market mainly cover the yellow-green wavelength range [5,6], and the near-infrared LEDs do not match well with the chlorophyll absorption band of plants because of their narrow luminous wavelength and low luminous intensity [7,8,9]. Therefore, broad, deep-red lighting devices suitable for plant growth have become the focus [10]. At present, phosphor-converted light-emitting diodes (pcLEDs) based on blue chips, are among the most effective lighting means [11,12,13,14].

They are energy efficient, ensure environmental protection, and a have long service life, a small size, and low costs. Red emission phosphors, such as (Sr, Ca)AlSiN_3_:Eu^2+^ [15], and K_2_TiF_6_:Mn^4+^ [16], have become commercially available to improve the color quality of white LEDs. However, their emission wavelengths cannot be tuned to the deep-red band and the strongest emission cannot be effectively absorbed by plants. In addition, the nitride synthesis conditions are harsh, rare-earth materials are expensive, and the mining/purification process of Eu^2+^ is harmful to the environment, making it expensive and less stable [17]. Further K_2_TiF_6_:Mn^4+^ phosphor cannot provide an effective absorption band of red light covered plants with broad spectra. Thus, how to achieve broad, deep-red phosphors that can be efficiently excited by blue light is a more important challenge [7].

Recently, materials doped with the transition-metal ion Cr^3+^ have been considered to be ideal red phosphors. Its 3d electrons are located in the outer layer and are very sensitive to the crystal environment [18,19,20,21,22]. Therefore, the selection of different matrices allows for tunable emission for Cr^3+^ from deep-red to NIR light by adjusting the surrounding crystal field environment. The Gd_3_Al_4_GaO_12_ garnet (GAGG) is a stable material for scintillators and phosphors that have a lower synthesis temperature compared to the commonly used garnets [23]. Karolina Elzbieciak and Lukasz Marciniak reported the strategy for modulating the relative sensitivity of Cr^3+^-based luminescent thermometers through substituting Al^3+^ ions with Ga^3+^ in Gd_3_Al_5−x_Ga_x_O_12_:Cr^3+^, Nd^3+^ and caused the gradual decline of the crystal field strength from Dq/B = 2.69 to Dq/B = 2.18, respectively, for Gd_3_Al_5_O_12_:Cr^3+^, Nd^3+^ and Gd_3_Ga_5_O_12_:Cr^3+^, Nd^3+^ [24]. Zhang et al. reported on broad-band near-infrared Ca_2_LuZr_2_Al_3_O_12_:Cr^3+^ garnet phosphor, which was used in combination with a 460 nm LED chip to fabricate pc-LED devices. Its photoelectric efficiency was 4.1%, which was better than that of tungsten lamps (2.9%) in the 750–820 nm spectrum range [25]. Cr^3+^ had the advantage of high efficiency and matching with blue LED chips compared to other NIR phosphors. However, the luminous efficiency needs further improvement.

Cr^3+^ luminescence can suffer from impurities and oxidation into Cr^4+^ when the materials are sintered in the air. As a result, the luminous efficiency of Cr^3+^ doped substrates reported so far has not been very high because of impurities. For instance, the external quantum efficiency (EQE) of Ca_3_Sc_2_Si_3_O_12_ (CSSG) was 12.8% [26]. The external quantum efficiency was increased to 21.5% by adding flux and sintering in a CO-reducing atmosphere [7]. In addition, rare-earth/Cr^3+^ co-doping appears to be a very promising method of improving luminous efficiency. For instance, a Ca_2_LuHf_2_Al_3_O_12_:Ce^3+^, Cr^3+^ sample synthesized using a conventional high-temperature solid-phase method is three times brighter than a single-doped Cr^3+^. Therefore, rare-earth/Cr^3+^ co-doping appears to be a very promising method of improving luminous efficiency [27].

In this paper, GAGG:Cr^3+^, Dy^3+^ samples were synthesized using a conventional high-temperature solid-phase method to obtain phosphors with high brightness and deep-red luminescence. The synthesis method is environment-friendly, simple, and cheap and leads to a pure Gd_3_Al_4_GaO_12_ phase. In GAGG:Cr^3+^, the absorption of Cr^3+^ comes from the d-d forbidden transition, its excitation efficiency is low. In order to obtain higher luminescence intensity, the sensitized ion Dy^3+^ was introduced into the GAGG:Cr^3+^ material. The energy transfer process between Cr^3+^ and Dy^3+^ in the GAGG is addressed. To the best of our knowledge, this is the first report detailing an energy transfer and the luminescent properties of a Cr^3+^-Dy^3+^ co-doped GAGG host. Moreover, this work represents an advance in the development and application of plant growth lighting. 

## 2. Experimental Section

A conventional high-temperature solid-phase method was used to synthesize GAGG:Cr^3+^, Dy^3+^ samples. The stoichiometries were Gd_3−*y*_Al_4−*x*_GaO_12_:*x*Cr^3+^, *y*Dy^3+^ with *x* = 0, 0.08, 0.1, 0.15, 0.2 and *y* = 0, 0.002, 0.006, 0.01, 0.014, 0.018. Gd_2_O_3_ (Aladdin, 99.99%) (Shanghai, China), Al_2_O_3_ (Aladdin, 99.9%), Ga_2_O_3_ (Aladdin, 99.99%), Cr_2_O_3_ (Aladdin, 99.99%), and Dy_2_O_3_ (Aladdin, 99.99%) were used as starting materials, and 0.07 mol of H_3_BO_3_ (Aladdin, 99.9%) was added as the flux. The above materials were weighed according to the stoichiometric ratio, grounded evenly in an agate mortar, mixed into corundum crucibles, and annealed at 1650 °C for 6 h in a box-type resistance furnace using a rate of 10 °C/min. The samples were protected by 5% H_2_/N_2_ gas flow during the whole sintering process. The calcined sample is naturally cooled to room temperature and grounded evenly to obtain a series of phosphor powders.

Powder X-ray diffraction was measured at room temperature using a PANalytical heaven Ⅱ diffractometer employing CuKα radiation. The scanning step was 0.02° in the range of 10–90° with 4 s per step integration. A scanning electron microscope (SEM, Hitachi S-3400-N, Homewood, AL, USA) was used to observe the product morphology. A fluorescence spectrophotometer (FS5, Edinburgh, Livingston, UK) equipped with a 450 W xenon lamp, was used to record the excitation and emission spectra of the samples and explore their luminescence performances. The thermal quenching test was completed using the FLS980 steady state transient fluorescence/phosphorescence spectrometer. The corresponding temperature-dependent emission properties of as-synthesized phosphors were measured on the FS5 fluorescence spectrometer. The temperature of the samples was controlled through an externally connected temperature controller (Orient KOJI, Hongkong, China). The samples were heated from 25 to 200 °C at a constant rate of 5 °C/min. The (EVERFINE) analysis system was used to test the packaged sample device.

## 3. Results and Discussion

### 3.1. Phase Identification and Crystal Structure

**Figure 1:**
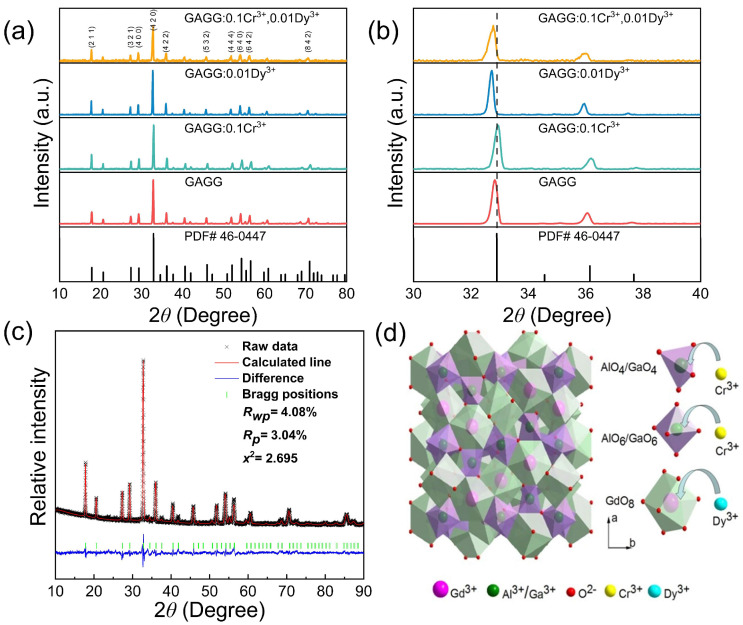
(**a**) XRD of GAGG, GAGG:Cr^3+^, GAGG:Dy^3+^ and GAGG:Cr^3+^, Dy^3+^; (**b**) magnified XRD patterns in the 30–40° range; (**c**) XRD refinements of GAGG; (**d**) crystal structure of GAGG viewed along the a-axis.

The XRD patterns of GAGG, GAGG:0.1Cr^3+^, and GAGG:0.1Cr^3+^, 0.01Dy^3+^ are shown in Figure 1a. These samples were basically consistent with the standard card (PDF # 46-0447). After Dy^3+^ doping, the XRD patterns shown in Figure 1b shifted toward smaller angles, which proved the successful doping of Dy^3+^. The shift was less pronounced after co-doping with Cr^3+^, indicating that the single Cr^3+^ doping or Cr^3+^ and Dy^3+^ co-doping had little effect on the matrix lattice parameters. The ionic radius of Cr^3+^ was 0.615 Å (CN = 6), Al^3+^ was 0.540 Å (CN = 6), Dy^3+^ was 0.912 Å (CN = 8) and Gd^3+^ was 0.938 Å (CN = 8); therefore, it is most likely that Dy^3+^ fits in the Gd^3+^ sites. Cr^3+^ is expected to emit near-infrared light in an octahedral rather than a tetrahedral environment, so Cr^3+^ prefers to replace Al^3+^ in an octahedral environment rather than Ga^3+^ in a tetrahedral environment [28,29,30]. All diffraction peaks were well indexed to GAGG, as shown in Figure 1a,c, and calculated using the Rietveld structure refinement method. Additionally, no extra peak appeared in the patterns, indicating that pure phase GAGG:Cr^3+^, Dy^3+^ phosphors had been achieved. Figure 1d shows the dodecahedral, octahedral, and tetrahedral positions. In the GAGG structure, the dodecahedral lattice (24c lattice) was occupied by Gd^3+^, and Al^3+^ and Ga^3+^ occupied octahedral and tetrahedral positions. However, when Cr^3+^ ions were doped into the system, they gave preference to octahedral coordination and then entered the tetrahedra.

**Figure 2:**
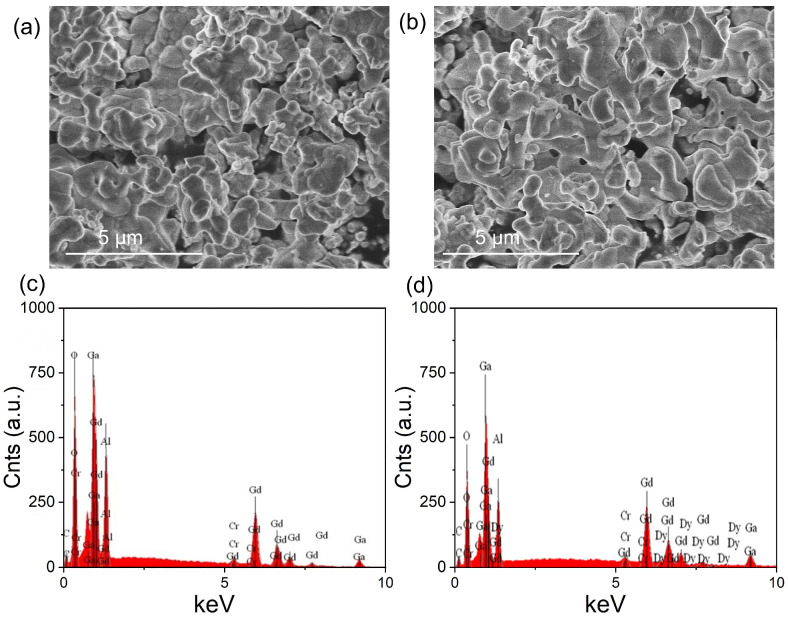
SEM photographs of GAGG:Cr^3+^ (**a**) and GAGG:0.1Cr^3+^, 0.01Dy^3+^ (**b**). EDS of GAGG:0.1Cr^3+^ (**c**) and GAGG:0.1Cr^3+^, 0.01Dy^3+^ (**d**).

Figure 2a,b presents SEM images of GAGG:0.1Cr^3+^ and GAGG:0.1Cr^3+^, 0.01Dy^3+^. EDX scanning was performed at 15 keV and 10 k magnification. Since the samples were ground, they showed an almost identical irregular morphology. Figure 2c,d shows the EDS energy spectra of GAGG: 0.1Cr^3+^ and GAGG:0.1Cr^3+^,0.01Dy^3+^. The EDS analysis confirmed that the samples contained Gd^3+^, Ga^3+^, O^2-^, Al^3+^, Cr^3+^ and Dy^3+^ as trace elements. XRD and SEM/EDS results, therefore, confirmed that Cr^3+^ and Dy^3+^ ions were successfully doped into the GAGG matrix.

### 3.2. Luminescence Properties

**Figure 3:**
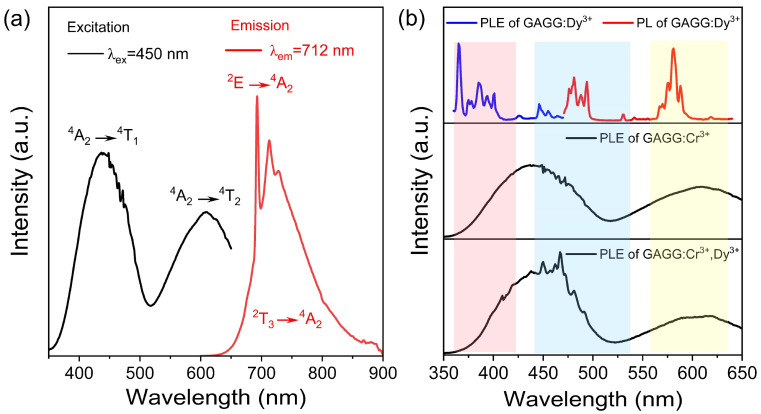
(**a**) PLE (λem = 712 nm) and PL (λex = 450 nm) spectra of GAGG:0.1Cr^3+^; (**b**) PL spectrum of GAGG:Cr^3+^ and GAGG:Cr^3+^, Dy^3+^ (black line), the PL (Blue line) and PLE spectra (red line) of GAGG:Dy^3+^.

The excitation and emission spectra of GAGG:Cr^3+^ are shown in Figure 3a. There are two excitation bands at 350–500 nm and 500–650 nm, which belong to the ^4^A_2_ → ^4^T_1_ and the ^4^A_2_ → ^4^T_2_ transitions of Cr^3+^, respectively. Under the 450 nm excitation, the characteristic Cr^3+^ emission composed of narrow peaks at 693 and 713 nm in the range of 650–850 nm, was observed. The emission from 650 to 850 nm originates from the ^4^T_2_ → ^4^A_2_ transition. The peak at 693 nm originates from the zero phonon line of the ^2^E → ^4^A_2_ spin forbidden transition (the R line) [31,32,33]. 

Typically, the Cr^3+^ sample shows a sharp line normally attributed to the spin-forbidden leap ^2^E → ^4^A_2_ [29]. The excitation spectra of GAGG:Cr^3+^ were mainly located in the blue light region, indicating that the GAGG:Cr^3+^ phosphor matches well with the emission of blue LED chips. The emission of GAGG:Cr^3+^ is located in the deep-red region and has a good overlap with the absorption spectrum of photosensitive pigments P_R_ and P_FR_. LEDs constructed using the GAGG:Cr^3+^ phosphor and blue chips could be ideal lighting devices for plant lighting.

The PLE (blue line) and PL (red line) spectra of GAGG:0.01Dy^3+^ are shown in Figure 3b. A wavelength of 575 nm was selected to detect the PLE spectrum, and a 450 nm wavelength was used to excite the sample. The excitation peaks at 352, 366, 387, 427, 452, and 476 nm were attributed to the Dy^3+^ transition from ^6^H_15/2_ to ^6^p_7/2_, ^6^p_5/2_, ^4^p_7/2_, ^4^G_11/2_, ^4^I_15/2_, and ^4^F_9/2_, respectively [34,35]. The emission peaks at 479 and 575 nm were attributed to the ^4^F_9/2_ → ^6^H_15/2_ and ^4^F_9/2_ → ^6^H_13/2_ transitions, respectively [36,37]. The PLE spectra show that both Cr^3+^ and Dy^3+^ can be excited by blue light at 450 nm. It is also found that the emission peak of Dy^3+^ overlapped with the excitation peak of Cr^3+^; thus, energy transfers in the Cr^3+^-Dy^3+^ co-doped sample were possible.

**Figure 4:**
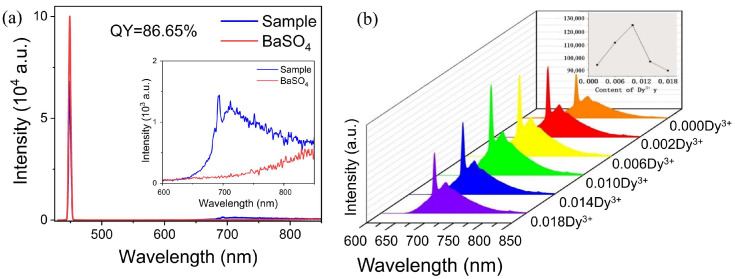
(**a**) Normalized spectra of the 450 nm light with BaSO_4_ as reference for quantum efficiency measurement and of GAGG:0.1Cr^3+^,0.01Dy^3+^; (**b**) emission spectra (λ_ex_ = 450 nm) of GAGG:0.1Cr^3+^, *y*Dy^3+^ (*y* = 0, 0.002, 0.006, 0.01, 0.014, 0.018).

The luminescence properties of Cr^3+^ and Dy^3+^ co-doped GAGG were further investigated, as shown in Figure 4. The content of Cr^3+^ was fixed at 0.1 mol, and the Dy^3+^ ion doping concentration changed from 0.002 to 0.18. For GAGG:0.1 Cr^3+^,0.01Dy^3+^, the luminescence intensity was the highest and the luminescence intensity was 1.4 times that of the Cr^3+^ single doped sample. At the same time, the internal quantum efficiency of GAGG:0.1 Cr^3+^,0.01Dy^3+^ reached the maximum of 86.65%. Even more interestingly, only the emission of Cr^3+^ was produced in GAGG:Cr^3+^,0.01Dy^3+^. Concentration quenching started to occur when the concentration of Dy^3+^ was greater than 0.01. This was probably caused by a total energy transfer from ^4^F_7/2_ toward ^4^T_2_ levels and to the E_g_ level that led to deep-red emission [25]. There was an energy transfer between Dy^3+^ and Cr^3+^. In addition, it was found that the addition of Dy^3+^ did not affect the luminescence peak position and waveform of Cr^3+^. Since the radius of Dy^3+^ (0.912 Å, CN = 8) was almost equal to that of Gd^3+^ (0.938 Å CN = 8), Dy^3+^ entered the lattice and only occupied the position of Gd^3+^, and the formed REO_8_ (RE = Gd, Dy) hardly affected the crystal field of the neighboring of CrO_6_ (GaO_6_) octahedrons [38].

### 3.3. Energy Transfer in GAGG: Cr^3+^, Dy^3+^

**Figure 5:**
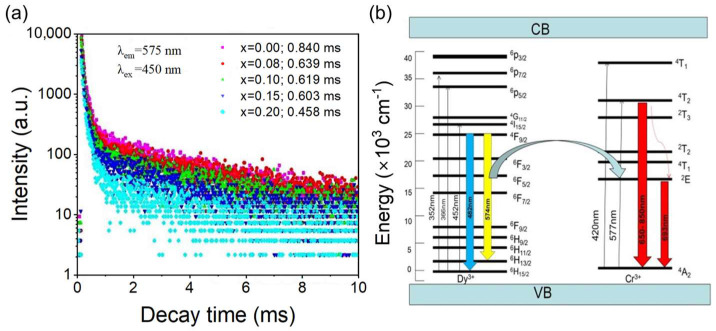
(**a**) Decay curves of Dy^3+^ emission in GAGG:*x*Cr^3+^, 0.01Dy^3+^; (**b**) energy levels, electron transitions and energy transfer schematic diagram of Dy^3+^ and Cr^3+^ in GAGG.

In order to further study the energy transfer between Dy^3+^ and Cr^3+^, the fluorescent decays of Dy^3+^ were measured, as shown in Figure 5a. The fluorescence attenuation curves of GAGG: *x*Cr^3+^, 0.01Dy^3+^ (*x* = 0, 0.08, 0.1, 0.15, 0.2) were measured under the 450 nm excitation and the 575 nm detection. The fluorescence attenuation curve was fitted using a second-order exponential attenuation model, with the formula as follows [39].

(1) $$I=I_{0}+A_{1}\exp(-t/\tau_{1})+A_{2}\exp(-t/\tau_{2})\;$$

A_1_ and A_2_ are the fitting constants, *I* is the intensity of fluorescence at time *t*, *τ*_1_ and *τ*_2_ are the fluorescence lifetime; The average attenuation-times are also fitted with a second-order index, as shown in Formula (2) [40].

(2) $$\tau =\left(A_{1}\tau_{1}^{2}+A_{2}\tau_{2}^{2}\right)/\left(A_{1}\tau_{1}+A_{2}\tau_{2}\right)$$

The fluorescence lifetime decreased from 0.84 to 0.458 ms with increasing Cr^3+^ concentration from 0 to 0.2 respectively. This result proves that energy transfers from Dy^3+^ to Cr^3+^ existed in these samples. We note a somewhat similar phenomenon in the Ca_14_(Al, Ga)_10_Zn_6_O_35_ matrix to what was reported by Zhou et al. [41].

The following equation can be used to calculate the energy transfer efficiency (η_ET_) [42].

(3) $$\eta_{\mathrm{ET}}=1-\tau_{s}/\tau_{s0\;}$$

where τ and τ_0_ are the lifetimes of Dy^3+^ with and without Cr^3+^. On the basis of the formula, the energy transfer efficiency increased from 23.9% to 45.48%.

To describe the energy change in GAGG:0.1Cr^3+^,0.01Dy^3+^ phosphor, the excitation, emission and energy transfer processes are shown in Figure 5b. Under the irradiation of 450 nm light, electrons are excited from the Dy^3+ 6^H_15/2_ energy level to the excited state, such as ^6^p_7/2_, ^6^p_5/2_, ^4^p_7/2_, ^4^G_11/2_, ^4^I_15/2_, and ^4^F_9/2_, and then relax to ^6^H_13/2_ and ^6^H_15/2_ from ^4^F_9/2_ with blue and orange emission. Meanwhile, electrons can also be excited into the Cr^3+ 4^T_1_ and then relax to the ^4^T_2_ and ^2^E energy levels, thus providing deep-red emission when relaxing to the ^4^A_2_ state. In this process, the energy transfer occurs from the excited state ^4^F_9/2_ of Dy^3+^ to the excited states ^4^T_2_ and ^2^E of Cr^3+^.

### 3.4. Temperature-Dependent Emission Spectra

**Figure 6:**
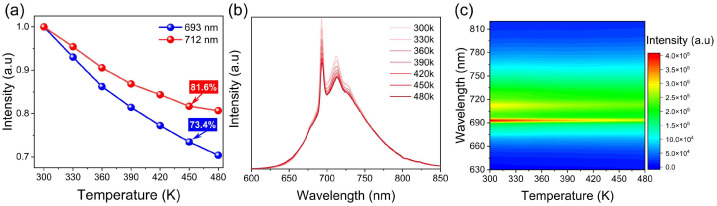
Emission intensities of 693 nm and 712 nm (**a**) and emission spectra; (**b**,**c**) of GAGG:0.1Cr^3+^, 0.01Dy^3+^ dependent on temperatures.

The normalized emission intensity, as a function of temperature is shown in Figure 6a. When the temperature is 440 K, the light intensities at 693 and 712 nm are 73.4% and 81.6%, respectively, of those at 300 K. Figure 6b illustrates the emission spectra of GAGG:0.1Cr^3+^, 0.01Dy^3+^ excited at 450 nm in the temperature range of 300–470 K. The profiles of the PL spectra do not experience major changes at different temperatures, while the intensity decreases with increasing temperatures owing to the thermal quenching effect [32]. Figure 6c shows the projection of the emission spectrum with increasing temperature, which shows the change of luminous intensity with increasing temperature. The increase in temperature leads to the intensification of lattice vibrations and an increase in the probability of non-radiative relaxations. The particles of each metastable state relax back to the ground state without radiation, and finally, the excitation energy is dissipated in the matrix lattice in the form of thermal energy. Compared with the spin-allowed transition from ^4^T_2_ (4F) to ^4^A_2_, the lattice vibration has a greater influence on the spin-forbidden ^2^E → ^4^A_2_ transition of Cr^3+^ [25,42].

### 3.5. LED Packages

**Figure 7:**
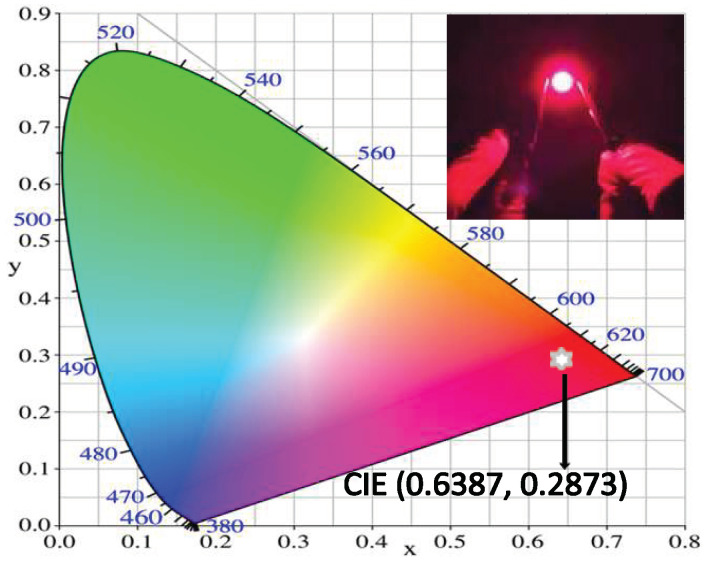
CIE chromaticity coordinates of the sample. The illustration shows the LED device in a 450nm chip package.

In order to demonstrate the applicability of the synthetic GAGG:0.1Cr^3+^, 0.01Dy^3+^ for indoor plant growth, LED devices were fabricated with the GAGG:0.1Cr^3+^, 0.01Dy^3+^ phosphor and a 450 nm blue chip. Figure 7 shows the resultant CIE coordinates of this LED device, which were found at (0.6387, 0.2873). It appears as a milky white light in the LED device and provides a bright purplish-red emission driven by a current of 20 mA. It gives a strong red emission and yields a luminous efficacy of 27.8 lmW^−1^. The results show that the new GAGG:0.1Cr^3+^,0.01Dy^3+^ phosphor can be excited by 450 nm of blue light and its red emission has a good overlap with the red light absorption of chlorophyll [8], demonstrating its potential for plant growth lighting and white LED lighting.

## 4. Conclusions

To sum up, Dy^3+^ and Cr^3+^ co-doped GAGG phosphors were successfully synthesized using a conventional high-temperature solid-state method. Dy^3+^ ions fit into Gd^3+^ sites and played the role of sensitizing the luminescence center for Cr^3+^. The luminescence intensity in deep-red light (650–850 nm) was enhanced by Dy^3+^/Cr^3+^ co-doping. The luminous intensity of optimized GAGG:Cr^3+^,0.01Dy^3+^ was 1.4 times that of the Cr^3+^ single-doped sample and its quantum efficiency was up to 86.65%. Many results point toward an energy transfer from Dy^3+^ to Cr^3+^ in GAGG:0.1Cr^3+^, 0.01Dy^3+^ phosphors. Finally, LED devices made from GAGG:0.1Cr^3+^, 0.01Dy^3+^ phosphors have good properties. This indicates potential applications of the phosphor in agriculture.

## Data Availability

Not applicable.
